# Collagen and Eosinophils in Jenny's Endometrium: Do They Differ With Endometrial Classification?

**DOI:** 10.3389/fvets.2020.00631

**Published:** 2020-09-10

**Authors:** Jordi Miró, Miguel Gutiérrez-Reinoso, Joana Aguiar da Silva, Carina Fernandes, Maria Rosa Rebordão, Graça Alexandre-Pires, Jaime Catalán, Graça Ferreira-Dias

**Affiliations:** ^1^Equine Reproduction Service, Department of Animal Medicine and Surgery, Faculty of Veterinary Medicine, Autonomous University of Barcelona, Cerdanyola del Vallès, Spain; ^2^Faculdade de Medicina Veterinária, CIISA - Centro de Investigação Interdisciplinar em Sanidade Animal, Universidade de Lisboa, Lisbon, Portugal; ^3^Coimbra College of Agriculture, Polytechnic Institute of Coimbra, Coimbra, Portugal

**Keywords:** donkey, endometrium, collagen, fibrosis, endometrosis, eosinophils, IL-33

## Abstract

Collagen fibers and inflammatory cells are the basis for jenny endometrium Kenney and Doig's classification developed for the mare. The infiltration of a large number of eosinophils in the jenny endometrium is intriguing. Eosinophil and fibroblast produced IL33, which has been related to fibrosis development and chronicity. This work on the endometrium consisted of (i) quantification of collagen type I (COL1A2), type III (COL3A1), and IL33 transcripts; (ii) histological localization and quantification of COL1 and COL3 proteins; and (iii) eosinophil and neutrophil count and correlation with collagen area and IL33 transcripts. Localization of COL protein in the jenny endometrium was also compared to the mare endometrium. As fibrosis increased, eosinophil and neutrophil count decreased (*P* < 0.05). A 5-fold increase in IL33 transcripts was noted from categories IIA to III. There was a tendency toward a positive correlation between eosinophil count and IL33 transcripts in category IIA endometrium (*P* = 0.055). Neither transcripts of COL1A2 nor COL3A1 nor the areas of COL1 or COL3 differed with endometrial categories. Unlike for the mare, and regardless of the jenny endometrium classification, COL3 was always found to different extents in the stratum compactum, while COL1 was mainly present in deep stroma. As fibrosis progressed in the mare, an extensive increase in COL1 fibers was notorious under the surface epithelium. Correlations between neutrophil count and COL1 and COL3 areas were observed in the jenny endometrium, although no correlation was found for eosinophil count. Neutrophil count positive correlation with the COL1 area and negative correlation with the COL3 area in endometria with mild lesions suggest that neutrophils in the jenny endometrium may be involved in fibrogenesis. In addition, when eosinophilia subsides, the endometrium reacts with fibrosis establishment, which could be stimulated by the pro-fibrotic cytokine IL33, whose release might then be ascribed to fibroblasts. Further studies are needed to analyze the effect of the presence of COL3 next to the surface epithelium in the stratum compactum, or around the endometrial glands on jenny's endometrial function and fertility.

## Introduction

In jenny's endometrium, the infiltration of eosinophils and the presence of fibrosis to different extents might influence endometrial function and gestation in a dissimilar fashion to the mare. The frequent infiltration of a large number of eosinophils in jenny's endometrium, unlike for the mare, appears to be a physiological feature of the jenny endometrium (1). The eosinophils appear to regulate tissue homeostasis and contribute for inflammatory reactions in tissue remodeling and innate immunity (2). In general, eosinophils are considered as the major effector cells in type two inflammatory diseases, including asthma, helminthic infection, and allergy (2). In fact, eosinophils result from the differentiation of CD117+ progenitor cells, directly stimulated by interleukin 33 (IL33) (3). In airway inflammation, IL33 besides exacerbating eosinophil infiltration in an autocrine and paracrine manner also attracts macrophages, lymphocytes, IL-13, and TGF-β1 (3). A relationship between eosinophil infiltration and the expression of IL33 has also been referred in chronicity and development of fibrosis in liver, skin, and intestine (4–6).

In the mare endometrium, the excessive deposition of collagen in the extracellular matrix, predominantly around the endometrial glands and stroma, which is associated with a chronic degenerative condition, destruction of tissue architecture, and impairment of endometrial function, is named endometriosis (7, 8). In tissue repair, collagen type III (COL3) is the first one to be expressed, being replaced by collagen type I (COL1) in later stages of fibrogenesis (9). Nevertheless, in the mare endometrium at the transcript level, age appeared not to influence COL1 or COL3 mRNA (10). In contrast, quantification of the areas in the endometrium occupied by COL1 and COL3, stained with histochemical picrosirius red staining and visualized by polarized light microscopy (11, 12), showed an increase in COL1 in the tissues with severe endometriosis, in comparison to healthy endometria or with mild fibrotic changes, where COL3 was the most predominant type (10). A previous morphometric analysis of periglandular fibrosis in equine endometria using picrosirius red also referred a predominance of COL1 in severe endometriosis (13).

The main purpose of this work was to contribute to the understanding of the inflammatory processes and fibrosis in jenny's endometrium, classified according to Kenney and Doig's mare grading system (14). Therefore, the main goals of the present study consisted of (i) quantification of transcripts of collagen type I (COL1A2), type III (COL3A1), and IL33 in the endometrium; (ii) localization and quantification of COL1 and COL3 protein in histological sections; and (iii) assessment of eosinophil and neutrophil count and their correlation with collagen and IL33 transcripts. In addition, localization of the COL protein in jenny's endometrium was compared to mare endometrium histological sections.

## Materials and Methods

### Animals and Sample Collection

A total of 80 Catalonian jennies, between 3 and 22 years of age, were divided in eight jenny groups, which were brought together for 75 days (April 1st/June 15th, 2017) with ten Catalan jackasses, aged 5–9 years and of proven fertility, for natural breeding. Jennies were assigned to different groups according to selection criteria. All animals were included in the Catalonian donkey Studbook and under a selection program. One month after jackass removal, a pregnancy diagnosis was done by transrectal ultrasonography using a MyLab™ Gamma (Esaote, Genova, Italy) device.

All animals were owned by a commercial donkey farm, FUIVES (Berga, Barcelona, Spain). Donkeys were kept in paddocks and fed grain forage, straw, hay, and water *ad libitum*.

A total of 29 jennies did not get pregnant (63.75% fertility rate), and 23 of them were included in the present study. Six jennies were discarded for different reasons: three of them presented cervical stenosis, one died, one showed a severe skin disease, and the other one had never foaled before. Endometrial biopsies were obtained during estrous from 23 non-pregnant jennies through the cervix, with an alligator jaw biopsy punch (Kevorkian's uterine biopsy forceps, Hauptner and Herberholz, Solingen, Germany), at estrus. All 23 jennies used in this study had foaled at least once before. Each biopsy was divided into two pieces. One portion was immediately immersed in buffered formaldehyde, for paraffin blocks, and further processed for histological examination. The other piece of the endometrium was placed in cryotubes with RNAlater® (AM7020; Ambion, Applied Biosystems, Foster, CA, USA) and kept at −80°C, for gene transcription studies, by real-time PCR.

### Histopathological Analysis

For histopathological analysis, 5-μm-thick sections of formaldehyde-fixed endometrium were stained with hematoxylin (05-06014E; Bio-Optica) and eosin (HT1103128; Sigma-Aldrich). Every jenny endometrial biopsy was evaluated by light microscopy (Leica DM500) regarding the histopathology of endometrial glands, presence of inflammatory cells (neutrophils, eosinophils), and fibrosis in the stroma. Neutrophil and eosinophil count was performed at random in 10 fields/biopsy, at the magnification of × 400. Due to the inexistence of a classification method for jenny's endometrium, the Kenney and Doig (14) grading system for the mare endometrium was used. Thus, a healthy endometrium was considered as category I; an endometrium with mild fibrotic lesions and inflammation as category IIA; when these lesions become moderate as category IIB; or when they are severe as category III (14).

### Real-Time PCR for COL1A2, COL3A1, and IL33

Quantification of COL1A2, COL3A1, and IL33 transcripts was accomplished by reverse transcriptase, followed by real-time polymerase chain reaction (qPCR), as previously described (15). Briefly, after endometrial total RNA extraction with TRI Reagent® (T9424; Sigma) according to the manufacturer's instructions, a NanoDrop system (ND200C; Fisher Scientific, Hampton, PA, USA) was used for RNA quantification. Visualization of 28S and 18S rRNA bands on a 1.5% agarose gel confirmed RNA quality. Reverse transcription was performed using M-MLV Reverse Transcriptase (M170B, Promega®) from 1 μg of total RNA in a 20-μL reaction volume using an oligo (dT) primer (C1101, Promega®).

Real-time PCR was used for the assessment of mRNA transcription of COL1A2, COL3A1, and IL-33. Glyceraldehyde-3-phosphate dehydrogenase (GAPDH) was chosen as the reference gene [<2-fold changes between different biological conditions; (16)] from four potential validated genes (ribosomal protein L32, succinate dehydrogenase A complex, subunit A, flavoprotein, and beta-2-microglobulin). Internet-based program Primer-3 (17) and Primer Premier softwares (Premier Biosoft Interpairs, Palo Alto, CA, USA) were used to design the primers listed in Table 1.

**Table 1:** Primer sequences used in real-time PCR analysis.

**Gene (Accession number)**	**Sequence 5′-3′**	**Amplicon (base pairs)**
COL1A2	Forward: CAAGGGCATTAGGGGACACA	196
(NM_001323780.1)	Reverse: ACCCACACTTCCATCGCTTC	
COL3A1	Forward: CAAAGGAGAGCCAGGAGCAC	98
(XM_014852914.1)	Reverse: CTCCAGGCGAACCATCTTTG	
GAPDH	Forward: CACCCACTCTTCCACCTTCG	173
(XM_014834961.1)	Reverse: CTTGCTGGGTGATTGGTGGT	
IL33	Forward: CCCCAGCAAAGATGAACAGC	179
(XM_014860749.1)	Reverse: TGGTACATGCCTTCTGGTGGT	

*COL1A2—Equus asinus collagen type I alpha 2 chain; COL3A1—Equus asinus collagen, type III, alpha 1; GAPDH—Equus asinus glyceraldehyde-3-phosphate dehydrogenase; IL33—Equus asinus interleukin 33*.

Target and reference gene amplification was performed on StepOnePlus™ Real-Time PCR System (Applied Biosystems®, California, USA) using Power SYBR Green PCR Master Mix (Applied Biosystems®), after primer concentration optimization. Polymerase chain reaction (PCR) products were run on a 2.5% agarose gel to confirm specificity. Results were evaluated using Real-time PCR Miner 4.0 software. For gene quantification, the average efficiency of each gene (E) and the average cyclic threshold (CT) of each sample were used, in the formula *r*_0_ = [1/(1 + E) CT] (18). Relative expression values were calculated by normalizing the expression level of the target genes against that of the reference gene.

### Picrosirius Red Staining

In order to assess the extent and localization of COL1 and COL3 in jenny's endometrium, a histological section, other than the one stained with hematoxylin and eosin for histopathological evaluation, was stained with Picrosirius Red Stain (ab#150681, Abcam plc, Cambridge, UK), following the manufacturer's instructions. Thus, a picrosirius red solution was placed onto deparaffinized endometrium sections and incubated for 1 h. Afterward, slides were rinsed with acetic acid solution and absolute alcohol. The slides were observed with an upright widefield microscope (Olympus BX51, Olympus Corporation, Tokyo, Japan) at 200× magnification (20×/0.75NA objective) under polarized light, and ten random images from each stained endometrium section were acquired with a camera (TIS 2MP RGB), with a pixel size of 0.441 μm/pixel (12). Collagen type one fibers appeared in red, while COL3 fibers stained green. A qualitative evaluation of the endometrium sections was performed to enable the identification of the areas where each type of collagen was present. Moreover, a quantitative analysis was performed with ImageJ program (Version 1.52a, National Institutes of Health, USA).

In order to better understand if the localization of collagen fibers, namely, COL1 and COL3, in jenny's endometrium was specific to this species and could be the possible explanation for their longevity in reproduction as compared to the mare, endometrial histological sections from mares, graded according to Kenney and Doig (14), were also stained with PSR and observed for a qualitative assessment.

### Statistical Analysis

Data regarding the area in the endometrial biopsy occupied by COL1 or COL3, and jenny's age in relation to endometrial category were statistically analyzed by one-way analysis of variance (ANOVA) followed by Tukey's *post hoc* test. In order to compare the number of eosinophils or neutrophils with IL33, COL1, or COL3 transcripts between endometrial categories, a Kruskal–Wallis analysis followed by Dunn's multiple-comparison test was used. Spearman rank correlation analysis between eosinophil or neutrophil count and IL33 mRNA levels and area occupied by COL1 or COL3 within each endometrial category was also performed. The software GraphPad Prism, Version 6.0, San Diego, CA, USA, was used to analyze these data. Significance was defined with the value of *P* < 0.05. Unless otherwise specified, the presented results are expressed as mean ± SEM.

## Results

### Histological Classification

**Figure 1:**
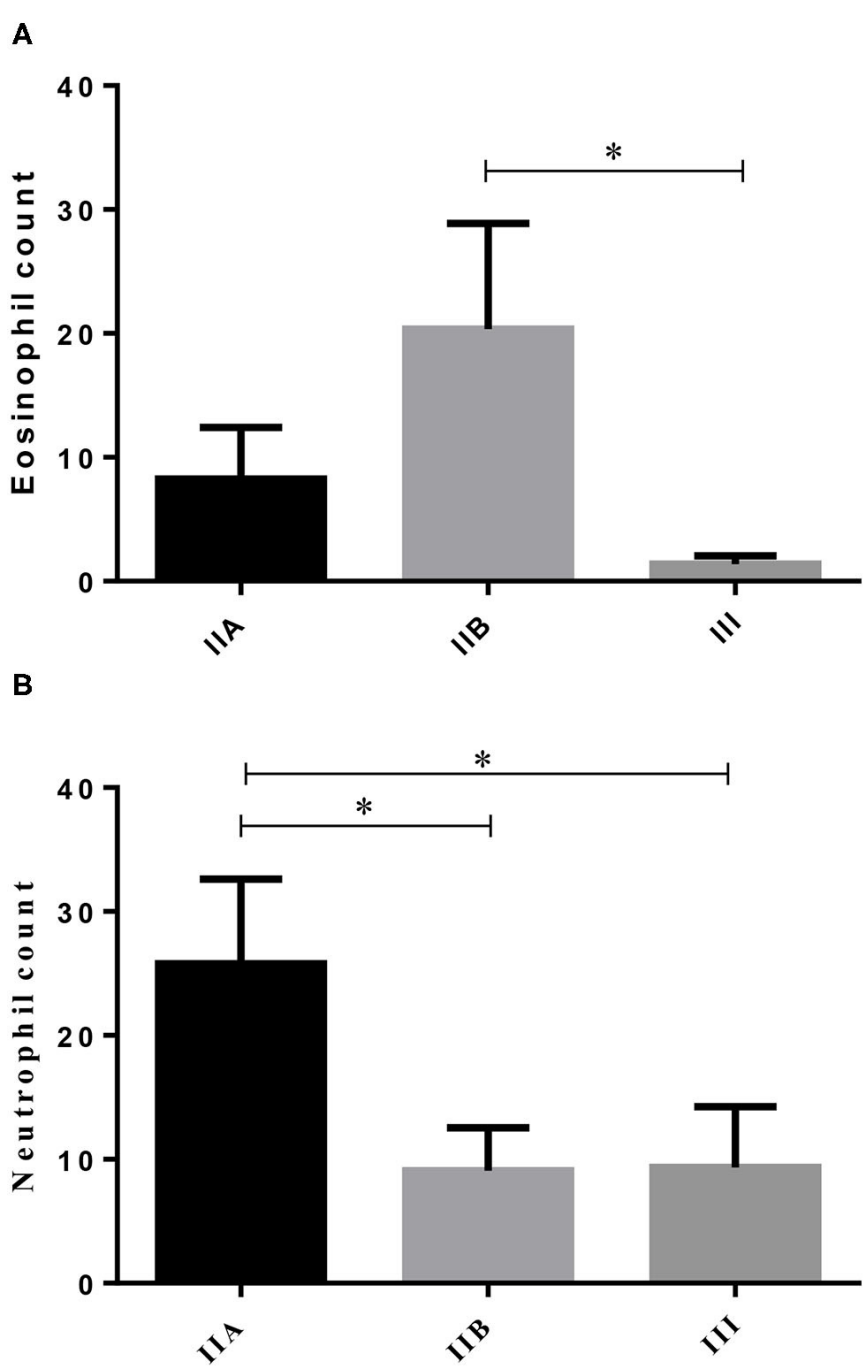
Eosinophil **(A)** and neutrophil **(B)** count in different endometrial categories (IIA, IIB, or III) of the jenny. Bars represent mean ± SEM. Asterisk indicates significant differences between endometrial categories (*P < 0.05).

From the 23 endometrial biopsies obtained from the jennies, six endometria were graded as category IIA (12.5 ± 3.9 years old), 11 as category IIB (13.8 ± 4.2 y), and six as category III (13.6 ± 2.2 y), according to Kenney and Doig's mare endometrial classification (14). No age differences were found among endometrial categories (*P* > 0.05). As fibrosis increased, from categories IIB to III, a significant 14-fold decrease in the number of eosinophils was depicted (Figure 1A; *P* < 0.05). Regarding neutrophil count, there was a fall from the category IIA endometrium to category IIB, and from categories IIA to III. No correlation was found between age and any of the studied factors (PMN, eosinophils, COL1, COL3, COL1, COL3, and IL33 transcripts) (Figure 1B; *P* > 0.05).

### Real-Time PCR

**Figure 2:**
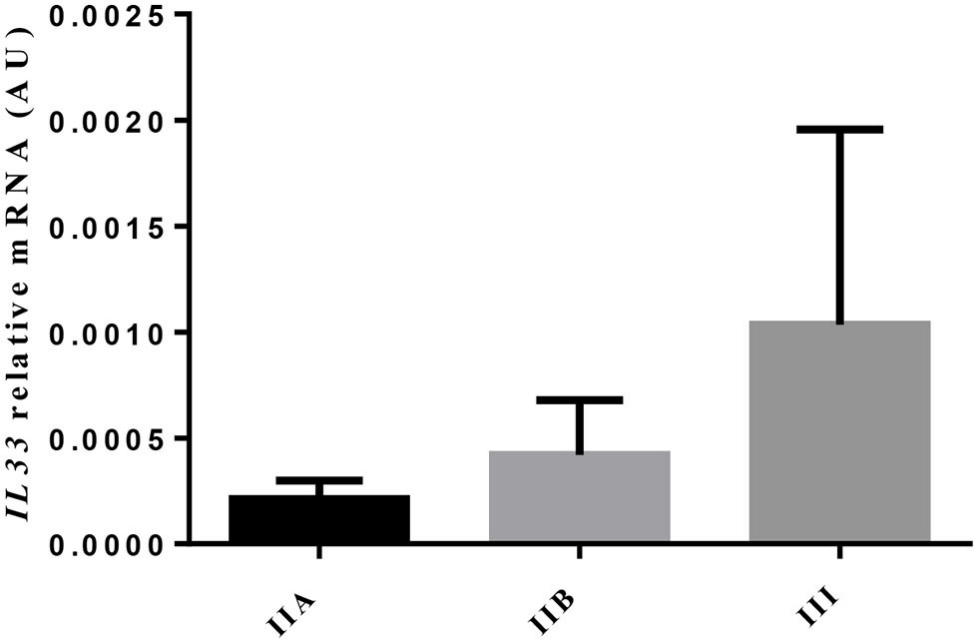
Relative IL33 mRNA levels in different endometrial categories (IIA, IIB, or III) of the jenny. Bars represent mean ± SEM.

The transcripts of IL-33, a cytokine involved in eosinophilia and in chronicity and development of fibrosis (6), showed no difference, as endometrial fibrosis advanced, from jenny's endometria with little collagen fibers, such as category IIA, to category III endometria, where fibrosis is visibly established (data not shown). Nevertheless, a 4.9-fold increase in IL33 mRNA levels was noted from category IIA to category III endometrium, even though not significant (Figure 2). When all endometrial categories were considered, no correlation was found between eosinophil count and IL33 transcripts (data not shown). However, there was a tendency toward a positive correlation between eosinophil count and IL33 transcripts in category IIA endometrium (ρ = 0.7714; *P* = 0.055), mainly due to the effect of a single sample (one out of six) that clearly tipped the line toward significance (data not shown).

**Figure 3:**
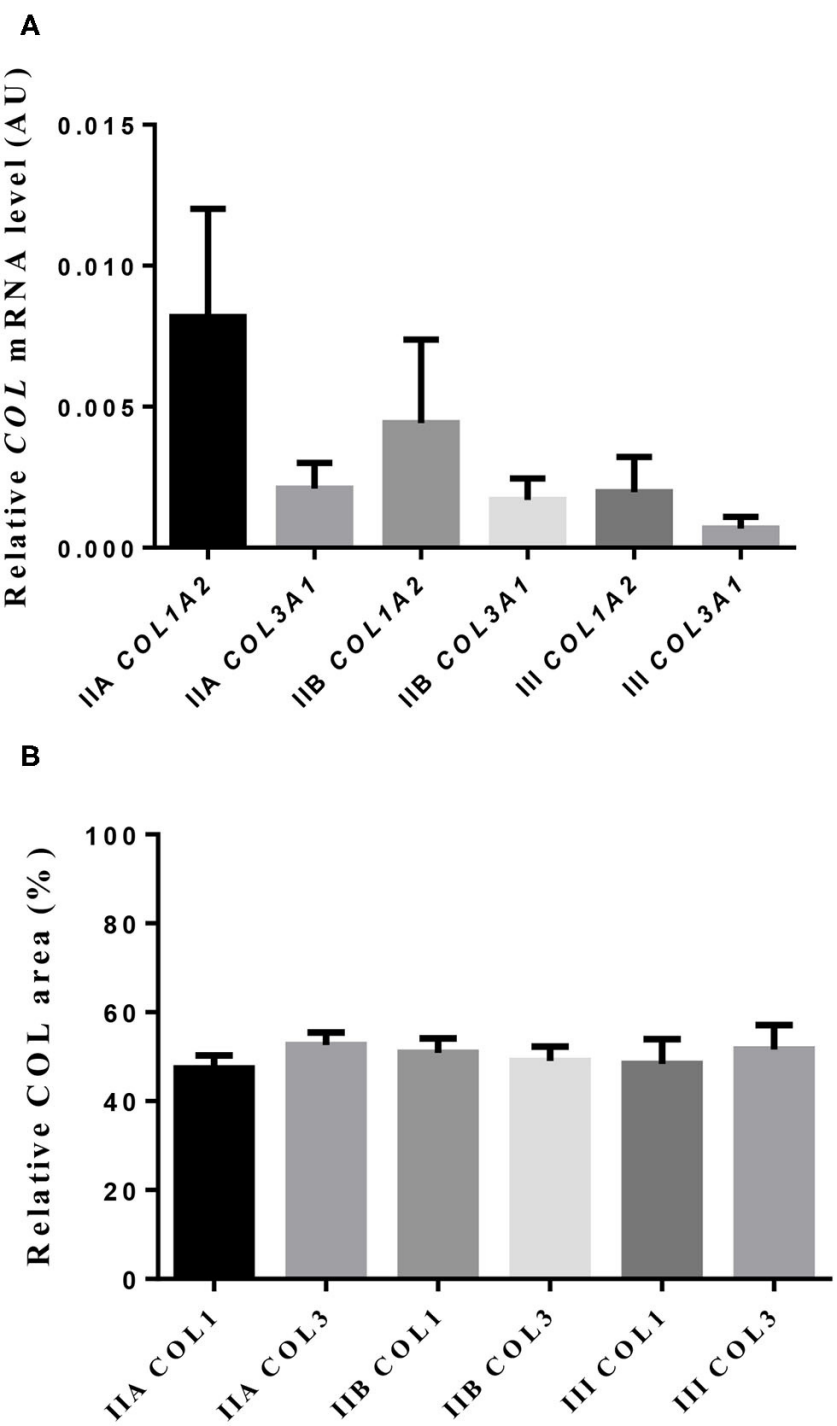
Relative mRNA levels **(A)** and proportion **(B)** of collagen type I (COL1) and type III (COL3) in jenny endometrium. Histologic sections were stained with picrosirius red. Bars represent mean ± SEM.

Neither transcripts of COL1A2 nor COL3A1 differed between jenny's endometrial categories (Figure 3A). However, a 4-fold drop in COL1A2 and a 3-fold fall in COL3A1 in the mRNA levels between category IIA and category III were noted.

### Picrosirius Red Staining

**Figure 4:**
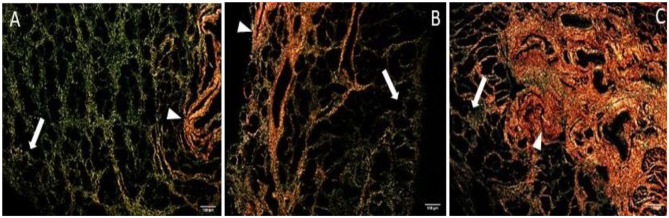
Jenny endometrium sections stained with picrosirius red and observed under polarized light microscopy. Collagen type I (COL1) stains red (arrow head), while collagen type III (COL3) stains green (white arrow). **(A)** Category IIA endometrium—a larger extent of COL3 is present in the stratum compactum under the surface epithelium and periglandular. Some COL1 fibers are present in the deeper stroma. **(B)** Category IIB endometrium—COL3 fibers are depicted under the basement membrane in the stratum compactum and periglandular, but COL3 fibers are also intertwined with some COL1 fibers. **(C)** Category III endometrium—COL3 fibers are still found under the basement membrane and periglandular, but more COL1 is present in the deep stroma, surrounding endometrial glands.

**Figure 5:**
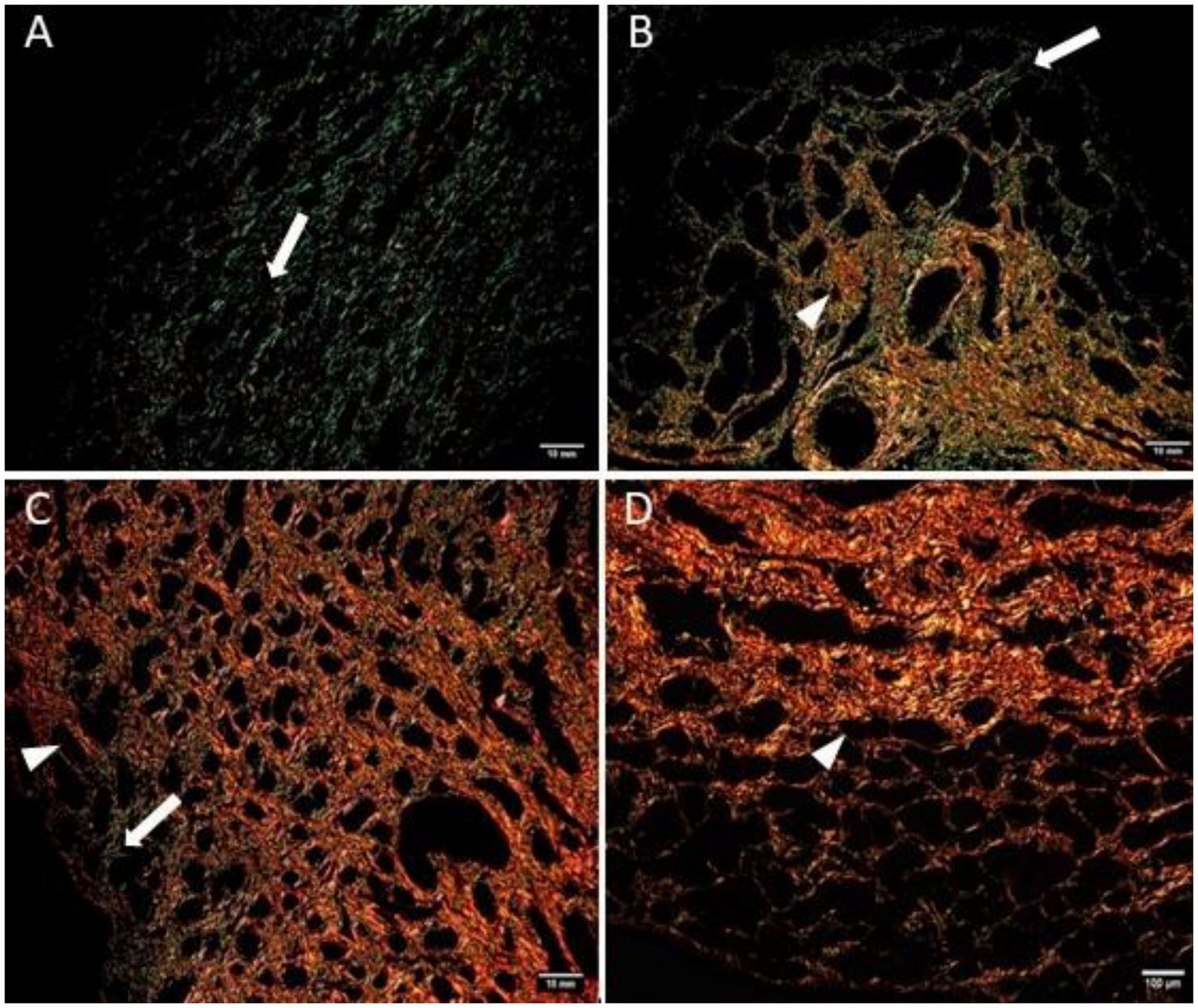
Mare endometrium sections stained with picrosirius red and observed under polarized light microscopy. Collagen type I (COL1) stains red (arrowhead), while collagen type III (COL3) stains green (white arrow). **(A)** Category I endometrium—a larger extent of COL3 is present in the stratum compactum under the surface epithelium and periglandular. **(B)** Category IIA endometrium—COL3 fibers are depicted under the basement membrane in the stratum compactum and periglandular, but COL3 fibers are also intertwined with some COL1 fibers. **(C)** Category IIB endometrium—COL3 fibers are sparsely found under the basement membrane, while COL1 is predominant and present in the deep stroma, surrounding endometrial glands. **(D)** Category III endometrium—COL1 is found almost exclusively in the stratum compactum, periglandular, and under the basement membrane.

The quantitative assessment of the different types of COL in the jenny endometrium has shown no differences in the area occupied by either COL1 or COL3 (Figure 3B). When considering the qualitative evaluation of collagen fibers in jenny's category IIA endometrium, green-stained COL3 was mostly found under the basement membrane and in the stroma, surrounding endometrial glands. However, COL1 fibers, which stained red, were sparse and mainly present in the deeper stroma (Figure 4, Table 2). In category IIB donkey endometrium, the location of COL3 fibers was also under the basement membrane in the stratum compactum and periglandular, but COL3 fibers were also intertwined with some COL1 fibers. In the stroma, both COL types were found. When fibrosis was strongly established, such as in category III endometrium, COL3 fibers were still found under the basement membrane and periglandular, but more COL1 was seen in the deep stroma, surrounding endometrial glands, lymphatic lacunae, and blood vessels, in comparison to the other Kenney and Doig's endometrial categories (Figure 4, Table 2). When these data were matched to mare's endometrial localization of COL fibers, visible differences were found between species. In mare endometria graded as category I (not found in the jennies in the present study), COL3 fibers were seen under the basement membrane and in the stratum compactum. As endometriosis progressed, an increase in COL1 fibers was notorious under the basement membrane, which was not seen in the jenny. Actually, in some category III endometria, only COL1 fibers were present (Figure 5, Table 3).

**Table 2:** Qualitative evaluation of the presence of collagen type I (COL1) and collagen type III (COL3) in jenny endometrium, stained with picrosirius red.

	**II A**	**II B**	**III**
Under basement membrane	COL3	COL3	COL3
Periglandular	COL3	COL3/COL1	COL3/COL1
Stroma	COL3	COL3/COL1	COL1 ↑
Deep stroma	COL1	COL1	COL1 ↑
Around lymphatic			COL1
Around blood vessels			COL1

**Table 3:** Qualitative evaluation of the presence of collagen type I (COL1) and collagen type III (COL3) in mare endometrium, stained with picrosirius red.

	**II A**	**II B**	**III**
Under basement membrane	COL3	COL3/COL1	COL1
Periglandular	COL3	COL3/COL1	COL3/COL1
Stroma	COL3	COL3/COL1	COL1 ↑
Deep stroma	COL1	COL1	COL1 ↑
Around lymphatic vessels			COL1
Around blood vessels			COL1

**Figure 6:**
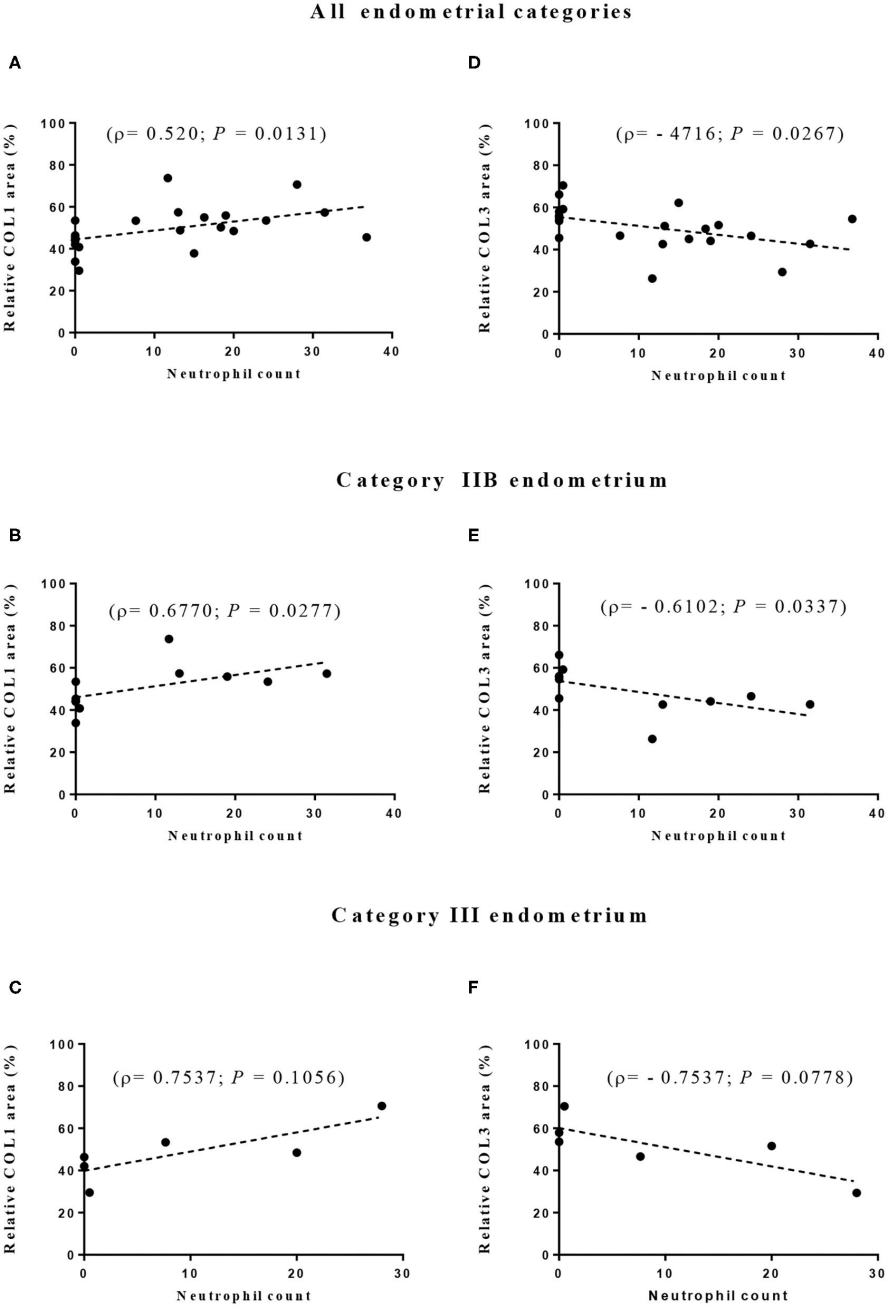
Correlation between neutrophil count and relative proportion of collagen type I (COL1—**A–C**) and type III (COL3—**D–F**) in all **(A,D)** and in IIB **(B,E)**, and III **(C,F)** jenny endometrial categories. ρ—Spearman rank correlation.

The correlation between eosinophil or neutrophil count and the area occupied by COL1 or COL3 was also evaluated. Although no correlation was found for eosinophil count (data not shown), correlations between neutrophil count and both COL1 and COL3 areas were observed. Positive correlations were seen for the endometrial area occupied by COL1 when all endometrial categories were considered (ρ = 0.520; *P* = 0.01; Figure 6A) and for category IIB (ρ = 0.6770; *P* = 0.03; Figure 6B) but not significantly in category III (Figure 6C). Between neutrophil count and COL3 area, a negative correlation was detected when all endometrial categories were considered (ρ = −0.4716; *P* = 0.03; Figure 6D) and in category IIB (ρ = −0.6102; *P* = 0.03; Figure 6E). A tendency toward a negative correlation between neutrophil count and COL3 area was also noticed in category III endometrium (ρ = −0.7537; *P* = 0.07; Figure 6F).

## Discussion

To the best of our knowledge, this is the first study that has evaluated IL33 expression in donkey endometrium and related its transcript levels with eosinophil count and collagen transcripts and deposition. Since IL33 is a critical regulator of a number of processes including inflammation, vascularization, and fibrosis (19, 20), its role on the progression of endometrial inflammation and fibrosis in the jenny is worth evaluating. In fact, IL33 can exacerbate inflammation in collagen-induced arthritis, and in allergic processes (21, 22). Based on studies on peritoneal fluid, serum, or samples from endometriotic lesions in women, it has been suggested that IL33 might play an important role toward inflammation present in advanced-stage endometriosis patients and on the progression of the disease (23, 24). Even though endometriosis in women, which consists of the abnormal growth of endometrial tissue outside the uterus (24, 25), and jenny's endometrial fibrosis are different conditions, at a certain stage of their development they both undergo an inflammatory process that ultimately may lead to fibrosis, as an end-point (25–29). We have shown that IL33 transcripts were detected in all jenny endometrial samples, regardless of their histopathological classification. Even though not reaching significance, IL33 mRNA levels in category III jenny endometrium were 5-fold increased, with respect to category IIA, while eosinophil count was about 14-fold lower in severe fibrosis than in healthier tissue (category IIA). The development of fibrosis and its chronicity in liver, skin, and intestine has been related to eosinophil infiltration and the expression of IL33 (4–6). Since a direct relationship between tissue eosinophil infiltration and the expression of IL33 has been referred, the present data on jenny endometria appears somewhat unexpected and cumbersome. In other species and tissues, accumulated observations have improved the understanding of eosinophil stimulation and ability to infiltrate tissues and participate in disease pathogenesis (30, 31). Even though rarely described, endometritis eosinophilica in the mare is a separate endometrial disease responsible for infertility (31). Nevertheless, deciphering the role of eosinophils in jenny's endometrium is still a challenge. It is plausible that jenny's endometrium with mild to moderate fibrosis (categories IIA and IIB) can mount an inflammatory reaction characterized by the presence of eosinophils and neutrophils. This eosinophil infiltration might be mediated by IL33. In fact, our results appear to indicate a trend toward a positive correlation between eosinophil count and IL33 transcripts in category IIA endometrium. Unlike other cytokines, when epithelial or endothelial cell damage or cell necrosis occurs, IL33 is passively released, suggesting that it may function as an alarmin that stimulates the immune system [reviewed by Chan et al. (22)]. However, in severe fibrosis (category III), as in other tissues where fibrosis is linked to impairment of organ function (32), the endometrial cells may no longer be capable of mounting an inflammatory reaction. Alternatively, eosinophil infiltration subsides, and the tissue reacts with fibrosis establishment, which could be stimulated by the still present pro-fibrotic cytokine IL33 (19), or by other pro-fibrotic stimuli (33). In spite of a decline in eosinophil infiltration in category III endometrium, the release of IL33 might be ascribed to other cells rather than eosinophils, such as fibroblasts [reviewed by Chan et al. (22)]. Nevertheless, this should be further investigated in jenny's endometrium.

In the present study, as fibrosis progressed from categories IIA to III, a 4-fold decrease in COL1A2 and a 3-fold decrease in COL3A1 in the mRNA levels were noted, although not significant. In addition, the percentage of areas occupied by COL1 and COL3 fibers in jenny endometria was similar, despite endometrial histopathological characteristics, in contrast to what we have previously shown for the mare endometrium. A preliminary work on Masson's trichrome-stained histological sections of jenny endometrium classified as I, IIA, or IIB was not conclusive about putative differences in the total area occupied by COL fibers (34). In contrast, in the mare, COL3 was the most predominant type of collagen in healthy endometrium, in comparison to severe endometriosis where COL1 was mostly found (10).

Interestingly, when evaluating the topographic histological distribution of COL1 and COL3 in jenny's endometrium, a different pattern was consistent, despite the histopathological classification and the progress of fibrosis. Even in category III endometrium, there was an area occupied by COL3 fibers under the basement membrane adjacent to the luminal epithelium, in the stratum compactum. In contrast, in the mare endometrium, COL3 fibers under the luminal epithelium were replaced by COL1, as fibrogenesis progressed. In fact, the arrangement of collagen fibers in the mare endometrium and their characteristics were related to the evolution of the process (35, 36). Mare foaling rates do not overcome 11% in category III endometrium (37). However, this correlation remains unknown in donkeys. The analysis of the Catalonian donkey stud book showed that only 3.3% of foaled jennies are older than 15 years of age^1^. Moreover, the generation interval index (average age of one jenny and its selected offspring) of this donkey breed is 7.75 ± 0.25 years^1^. However, based on these data, it is impossible to calculate jenny's reproductive longevity or overall fertility rate, let alone for each endometrial category. In addition, it has been referred that in mare endometriotic tissue, the incidence of periglandular fibrosis should not be based on the increased presence of collagen fibers (38). Instead, the arrangement of fibroblasts around endometrial glands, in one or more layers, which produce the extracellular matrix proteins such as collagen IV, laminin, and fibronectin, should be taken into consideration (38).

The connection between inflammation and fibrosis is well-recognized (31). One putative link between neutrophil and fibrogenesis has been provided by neutrophil extracellular traps (NETs, released by neutrophils) triggering the differentiation of myofibroblasts, cells responsible for COL deposition (17). In the mare, NET components induced an *in vitro* increase in COL1 in the endometrium (39). Likewise, in mares with closed cervix, a marked endometritis and intense neutrophil infiltration, and permanent pathological endometrial changes, including fibrosis were observed (40). In a recent study on jenny's endometrial cytology, an increase in neutrophil count in older and multiparous jennies was referred (41). In the present study, the neutrophil count positive correlations with the area occupied by COL1, and the negative correlation with the area of COL3 observed in endometria with mild lesions, suggest that the presence of neutrophils in jenny's uterus may be involved in endometrial fibrogenesis.

In conclusion, there are important differences in the collagen distribution between jenny and mare endometria. The presence of COL3 next to the surface epithelium, in the stratum compactum, and around endometrial glands might not have a deleterious effect on implantation and endometrial function, as it appears to occur in mares with category III endometrium. Further studies are needed to correlate these findings with jenny's fertility and age Therefore, donkey endometrium classification should be reconsidered. In addition, further studies are needed to unravel the unknown role of neutrophils and eosinophils present in jenny's endometrium on the reproductive performance of this species.

## Data Availability Statement

The raw data supporting the conclusions of this article will be made available by the authors, without undue reservation.

## Ethics Statement

Written informed consent was obtained from the owners for the participation of their animals in this study.

## Author Contributions

JM and GF-D conceived the study, participated in the sample acquisition and analysis, helped conduct the data analysis, contributed to the writing of the original draft, and reviewing and editing the manuscript. MG-R and JC participated in the jenny control, sample acquisition, and process and data curation. JS, CF, and GA-P performed the sample formal analysis, data curation, and analysis. MR participated in sample formal analysis, data curation, and analysis and writing of the original draft. All authors contributed to the article and approved the submitted version.

## Conflict of Interest

The authors declare that the research was conducted in the absence of any commercial or financial relationships that could be construed as a potential conflict of interest.

## Footnotes

^1^Rac.uab.cat/enllacos/racenlla.htm

## References

[1] Vilés K Rabanal R Rodríguez-Prado M Miró J. Effect of ketoprofen treatment on the uterine inflammatory response after AI of jennies with frozen semen. Theriogenology. (2013) 79:1019–26. 10.1016/j.theriogenology.2013.01.006 23453786

[2] Rothenberg ME Hogan SP. The eosinophil. Annu Rev Immunol. (2006) 24:147–74. 10.1146/annurev.immunol.24.021605.090720 16551246

[3] Gervasi MG Osycka-Salut C Caballero J Vazquez-Levin M Pereyra E Billi S. Anandamide capacitates bull spermatozoa through CB1 and TRPV1 activation. PLoS ONE. (2011) 6:e16993. 10.1371/journal.pone.0016993 21347292 PMC3037938

[4] Mchedlidze T Waldner M Zopf S Walker J Rankin AL Schuchmann M. Interleukin-33-dependent innate lymphoid cells mediate hepatic fibrosis. Immunity. (2013) 39:357–71. 10.1016/j.immuni.2013.07.018 23954132 PMC4172965

[5] Rankin AL Mumm JB Murphy E Turner S Yu N McClanahan TK. IL-33 induces IL-13–dependent cutaneous fibrosis. J Immunol. (2010) 184:1526–35. 10.4049/jimmunol.0903306 20042577

[6] Masterson JC Capocelli KE Hosford L Biette K McNamee EN De Zoeten EF. Eosinophils and IL-33 perpetuate chronic inflammation and fibrosis in a pediatric population with stricturing crohn's ileitis. Inflamm Bowel Dis. (2015) 21:2429–40. 10.1097/MIB.0000000000000512 26218140 PMC4567482

[7] Kenney RM The aetiology, diagnosis and classification of chronic degenerative endometritis (CDE) (endometriosis). In: Allen WR, editor. Equine Endometritis Worship. Proceedings of the John P. Hughes International Workshop on Equine Endometritis; 1992 Aug 19-20. Newmarket (1993). 10.1111/j.2042-3306.1993.tb02940.x

[8] Hoffmann C Ellenberger C Mattos RC Aupperle H Dhein S Stief B. The equine endometrosis: new insights into the pathogenesis. Anim Reprod Sci. (2009) 111:261–78. 10.1016/j.anireprosci.2008.03.019 18468817

[9] Bochsler PN Slauson DO Inflammation and repair of tissue. In: Slauson DO Cooper BJ, editors. Mechanisms of Disease: A Textbook of Comparative General Pathology. St. Louis, MO: Mosby (2002). p. 140–245.

[10] Pinto-Bravo P Reborddo MR Amoral A Fernandes C Cuello C Parrilla I Is mare endometrosis linked to oviduct fibrosis? Pferdeheilkunde. (2018) 34:43–6. 10.21836/PEM20180107

[11] Junqueira LCU Cossermelli W Brentani R. Differential staining of collagens type I, II and III by sirius red and polarization microscopy. Arch Histol Jap. (1978) 41:267–74. 10.1679/aohc1950.41.267 82432

[12] Podico G Canisso IF Roady PJ Austin SM Carossino M Balasuriya U. Uterine responses and equine chorionic gonadotropin concentrations after two intrauterine infusions with kerosene post early fetal loss in mares. Theriogenology. (2020) 147:202–10. 10.1016/j.theriogenology.2019.11.014 31787468

[13] Porto CD Caracterização histoquímica do colágeno e expressão de MMP-2, MMP-9 e TIMP-1 nas endometrites crônicas das éguas (Dissertation/Master's thesis). Botucatu: University Estadual Paulista “Júlio de Mesquita Filho” (2006).

[14] Kenney R Doig PA Equine endometrial biopsy. In: Morrow DA, editor. Current Therapy in Theriogenology. Philadelphia, PA: WB Saunders (1986). p. 723–9.

[15] Rebordão MR Amaral A Lukasik K Szóstek-Mioduchowska A Pinto-Bravo P Galvão A. Impairment of the antifibrotic prostaglandin E 2 pathway may influence neutrophil extracellular traps–induced fibrosis in the mare endometrium. Domest Anim Endocrinol. (2019) 67:1–10. 10.1016/j.domaniend.2018.10.004 30522057

[16] Dheda K Huggett JF Bustin SA Johnson MA Rook G Zumla A. Validation of housekeeping genes for normalizing RNA expression in real-time PCR. Biotechniques. (2004) 37:112–9. 10.2144/04371RR03 15283208

[17] Chrysanthopoulou A Mitroulis I Apostolidou E Arelaki S Mikroulis D Konstantinidis T. Neutrophil extracellular traps promote differentiation and function of fibroblasts. J Pathol. (2014) 233:294–307. 10.1002/path.4359 24740698

[18] Zhao S Fernald RD. Comprehensive algorithm for quantitative real-time polymerase chain reaction. J Comput Biol. (2005) 12:1047–64. 10.1089/cmb.2005.12.1047 16241897 PMC2716216

[19] Gao Q Li Y Li M. The potential role of IL-33/ST2 signaling in fibrotic diseases. J Leukoc Biol. (2015) 98:15–22. 10.1189/jlb.3RU0115-012R 25881899

[20] Millar NL O'Donnell C McInnes IB Brint E. Wounds that heal and wounds that don't – the role of the IL-33/ST2 pathway in tissue repair and tumorigenesis. Semin Cell Dev Biol. (2017) 61:41–50. 10.1016/j.semcdb.2016.08.007 27521518

[21] Liew FY. IL-33: a janus cytokine. Ann Rheum Dis. (2012) 71:101–4. 10.1136/annrheumdis-2011-200589 22460136

[22] Chan BCL Lam CWK Tam LS Wong CK. IL33: roles in allergic inflammation and therapeutic perspectives. Front Immunol. (2019)10:364. 10.3389/fimmu.2019.00364 30886621 PMC6409346

[23] Santulli P Borghese B Chouzenoux S Vaiman D Borderie D Streuli I. Serum and peritoneal interleukin-33 levels are elevated in deeply infiltrating endometriosis. Hum Reprod. (2012) 27:2001–9. 10.1093/humrep/des154 22587998

[24] Miller JE Monsanto SP Ahn SH Khalaj K Fazleabas AT Young SL. Interleukin-33 modulates inflammation in endometriosis. Sci Rep. (2017) 7:1–11. 10.1038/s41598-017-18224-x 29263351 PMC5738435

[25] Bulun SE. Endometriosis. N Engl J Med. (2009) 360:268–79. 10.1056/NEJMra0804690 19144942

[26] Itoga T Matsumoto T Takeuchi H Yamasaki S Sasahara N Hoshi T. Fibrosis and smooth muscle metaplasia in rectovaginal endometriosis. Pathol Int. (2003) 53:371–5. 10.1046/j.1440-1827.2003.01483.x 12787311

[27] Vicino M Scioscia M Resta L Marzullo A Ceci O Selvaggi LE. Fibrotic tissue in the endometrioma capsule: surgical and physiopathologic considerations from histologic findings. Fertil Steril. (2009) 91:1326–8. 10.1016/j.fertnstert.2008.02.157 18410939

[28] Sokkar SM Hamouda MA El-Rahman SM. Endometritis in she donkeys in Egypt. J Vet Med B Infect Dis Vet Public Health. (2001) 48:529–36. 10.1046/j.1439-0450.2001.00457.x 11666035

[29] Canisso IF Panzani D Miró J Ellerbrock RE. Key aspects of donkey and mule reproduction. Vet Clin North Am Equine Pract. (2019) 35:607–42. 10.1016/j.cveq.2019.08.014 31672204

[30] Akuthota P Weller PF. Eosinophils and disease pathogenesis. Semin Hematol. (2012) 49:113–9. 10.1053/j.seminhematol.2012.01.005 22449621 PMC3571705

[31] Grimm AL Schoon H-A Schöniger S. Histopathological features of endometritis eosinophilica in mares. Histol Histopathol. (2017) 32:1161–73. 10.14670/HH-11-872 28105630

[32] Zeisberg M Kalluri R. Cellular mechanisms of tissue fibrosis. 1. Common and organ-specific mechanisms associated with tissue fibrosis. J Physiol Cell Physiol. (2013) 304:216–25. 10.1152/ajpcell.00328.2012 23255577 PMC3566435

[33] Sziksz E Pap D Lippai R Béres NJ Fekete A Szabó AJ. Fibrosis related inflammatory mediators: role of the IL-10 cytokine family. Mediators Inflamm. (2015) 2015:764641. 10.1155/2015/764641 26199463 PMC4495231

[34] Pires MA Ferreira-Dias G Catarino J Bastos-de-Carvalho C Silvestre M Nóvoa M Correlation between morphological characterization of jenny (*Equus asinus*) endometrial biopsy and quantification of collagen deposition by image analysis. J Comp Pathol. (2019) 166:139 10.1016/j.jcpa.2018.10.125

[35] Masseno APB Assessment of endometrial fibrosis and myofibroblasts in endometriosis active and inactive mares (Dissertation/Ph.D. thesis). Botucatu: University Estadual Paulista “Júlio de Mesquita Filho” (2012).

[36] Costa LD Histochemical and immunohistochemical characterization of fibrotic changes in mares endometrosis (Dissertation/Master's thesis). Botucatu: University Estadual Paulista “Júlio de Mesquita Filho” (2015).

[37] Kenney RM. Cyclic and pathologic changes of the mare endometrium as detected by biopsy, with a note on early embryonic death. J Am Vet Med Assoc. (1978) 172:241–62. 621166

[38] Walter I Handler J Reifinger M Aurich C. Association of endometriosis in horses with differentiation of periglandular myofibroblasts and changes of extracellular matrix proteins. Reproduction. (2001) 121:581–6. 10.1530/reprod/121.4.581 11277878

[39] Rebordão MR Amaral A Lukasik K Szóstek-Mioduchowska A Pinto-Bravo P Galvão A. Constituents of neutrophil extracellular traps induce *in vitro* collagen formation in mare endometrium. Theriogenology. (2018) 113:8–18. 10.1016/j.theriogenology.2018.02.001 29452855

[40] Reilas T Rivera del Alamo MM Liepina E Yeste M Katila T. Effects on the equine endometrium of cervical occlusion after insemination. Theriogenology. (2016) 85:617–24. 10.1016/j.theriogenology.2015.09.053 26586278

[41] Quartuccio M Cristarella S Medica P Fazio E Mazzullo G Rifici C. Endometrial cytology during the different phases of the estrous cycle in jennies: new evidences. Animals. (2020) 10:1062. 10.3390/ani10061062 32575538 PMC7341222

